# Ovariohysterectomy and Partial Vaginectomy for Treatment of Cervicovaginitis in a Dog

**DOI:** 10.1155/2019/1357624

**Published:** 2019-12-23

**Authors:** Christian A. Folk, Cassie N. Lux, Whitney DeGroot

**Affiliations:** ^1^Veterinary Specialty Hospital, San Diego, CA, USA; ^2^The University of Tennessee College of Veterinary Medicine, Knoxville, TN, USA; ^3^Veterinary Emergency Clinic, Toronto, Ontario, Canada

## Abstract

A 1-year-old sexually intact female Labrador Retriever was evaluated for malodorous vaginal discharge, lethargy, and vomiting. A diagnosis of pyometra was suspected based on signalment, clinical signs, and abdominal ultrasonography. The dog underwent an exploratory celiotomy revealing a palpably enlarged cervix and edematous, fluid-filled vagina with an otherwise normal uterus. The ovaries, uterus, cervix, and cranial vagina were surgically resected. Histopathology revealed mild to moderate regionally extensive subacute neutrophilic cervicovaginitis due to an unknown underlying etiology. The dog did not exhibit any postoperative complications or recurrence of clinical signs in 6 months. This case represents an unusual disease condition, which presented in a manner typical for pyometra, yet required more extensive surgical resection.

## 1. Introduction

Vaginitis, or inflammation of the vagina, is a rare disease in dogs that is generally considered to be primary, secondary, or age-related and in most cases, is often self-limiting [1–6]. The treatment for vaginitis, when necessary, consists of systemic antibiotic therapy, oral probiotics, vaginal cleaning with an antiseptic, and/or surgical correction of predisposing anatomical abnormalities [1, 3, 4, 7]. While surgical intervention for vaginitis is uncommon and often not required, subtotal vaginectomy has been described for the treatment of extensive chronic vaginitis in a dog [8]. The case presented here describes diagnostic imaging findings of cervicovaginitis and partial vaginectomy for treatment of regionally extensive subacute neutrophilic cervicovaginitis, which to the author's knowledge has previously been unreported.

## 2. Case Description

A one-year-old 29.1-kg (64.0-lb) sexually intact female Labrador Retriever was presented to an emergency and referral veterinary hospital for a 3-day history of brown, malodorous vaginal discharge, lethargy, and a single episode of vomiting. The dog was noted to be undergoing her first heat cycle ten days prior to presentation characterized by intermittent hemorrhagic, odorless vaginal discharge which progressed to brown, malodorous discharge over a week.

On physical examination, the dog was febrile (40°C [104°F]) and around 5–7% dehydrated based on prolonged skin tent and dry mucous membranes. The dog's vulva appeared swollen with the presence of brown, malodorous vaginal discharge. In-house cytologic examination of the vaginal discharge was performed, which revealed neutrophilic inflammation with intracellular bacteria. Abdominal palpation revealed a tense abdomen, most notably in the caudal abdomen. Results of a complete blood count (CBC) revealed a moderate neutropenia (1.9 × 10^3^/uL; reference range, 2.41–10.88 × 10^3^/uL). A serum biochemical analysis revealed unremarkable findings.

**Figure 1:**
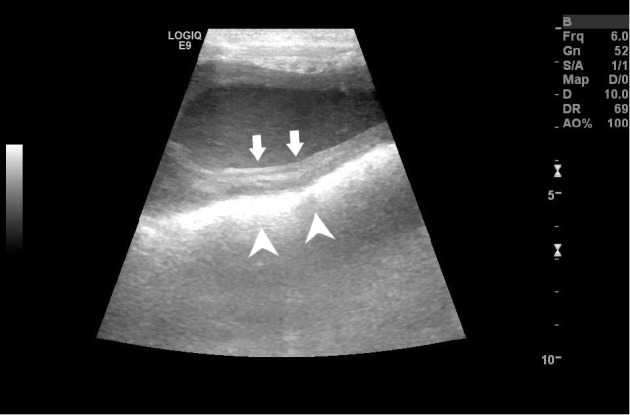
Longitudinal B-mode ultrasonographic view of the caudal aspect of the proximal vagina of a 1-year-old sexually intact female Labrador Retriever that was evaluated for a 4-day history of brown, malodorous vaginal discharge, vomiting, and a fever. Notice the echogenic fluid present within the vaginal lumen. The vaginal wall appears moderately thickened (arrows), which is just ventral to the hyperechoic region denoting fecal material within the colon (arrowheads). The image was obtained transabdominally with a 6-MHz linear transducer.

**Figure 2:**
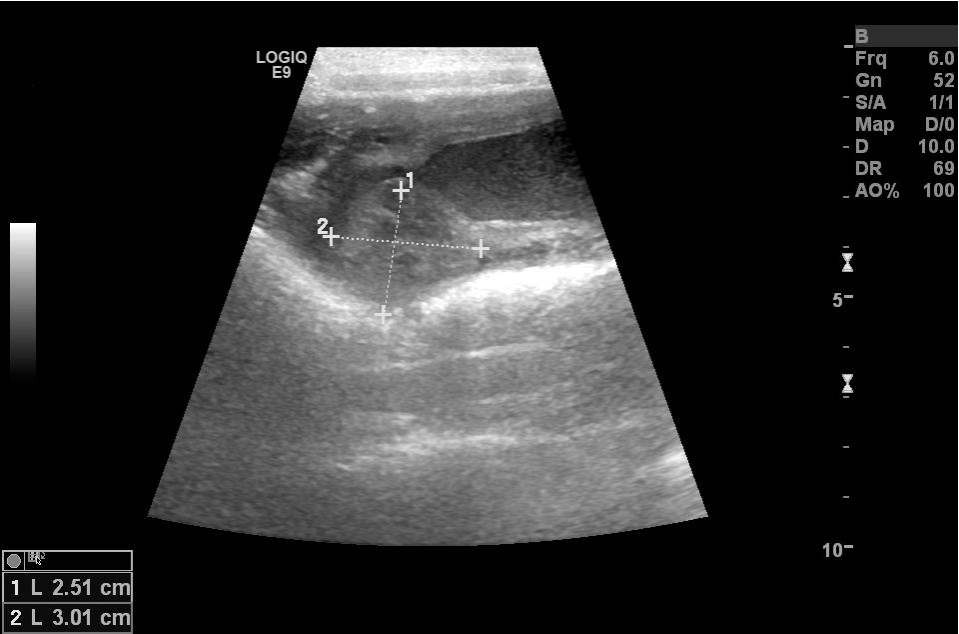
Longitudinal B-mode ultrasonographic view of the cranialaspect of the proximal vagina of a 1-year-old sexually intact female Labrador Retriever that was evaluated for a 4-day history of brown, malodorous vaginal discharge, vomiting, and a fever. Notice the focal thickening of the caudodorsal aspect of the cervix measuring 2.5 × 3 cm (plus signs). The image was obtained transabdominally with a 6-MHz linear transducer.

Abdominal ultrasonography revealed a tubular structure with a thickened wall containing echogenic fluid within its lumen in the caudal abdomen just dorsal and slightly cranial to the apex of the urinary bladder and ventral to the colon (Figure 1). It was initially suspected that this tubular organ was the uterine body, as it can usually be found just cranial and dorsal to the trigone of the bladder [9–11]. Continued ultrasonographic examination of this area revealed a heterogeneous, well defined, intramural mass like protrusion measuring 2.5 × 3 cm extending ventrally into the lumen of the presumed uterine body (Figure 2). Given the diagnostic imaging findings and initial primary differential diagnosis of suspected pyometra, the decision was made to perform an exploratory laparotomy at the referring veterinary practice. Abdominal exploratory revealed the ovaries, uterine horns, and uterine body to be grossly normal in appearance. Continued examination of the reproductive tract affirmed an apparent, significantly enlarged cervix caudal to the uterus. Due to the abnormal anatomy findings, the referring veterinarian closed the abdominal cavity without performing any additional surgical procedures. The dog was referred to the author's institution for further assessment and additional surgical consultation. Initiation of antibiotic therapy with amoxicillin-clavulanic acid (20 mg/kg, PO, *q* 12 hr) was started at the time of discharge by the referring veterinarian.

Upon arrival to the author's institution, the physical examination findings, abdominal ultrasound images, case history, and intraoperative photos of the dog were reviewed. Given the dog's significant fever, neutropenia, and vaginal discharge, it was suspected the dog had an atypical form of an open pyometra. The ultrasound images were not definitive of a pyometra, as the tubular, fluid-filled structure with thickened walls ultrasonographically was very caudal and suspected to be the distal uterine body, cervix, or proximal portion of the dog's vagina. The proximal part of the canine vagina can be visualized with abdominal ultrasonography, but often requires the instillation of saline into the vaginal vault for adequate visualization [10].The intramural mass like protrusion measuring 2.5 × 3 cm extending into the cranial vaginal lumen also seen with abdominal ultrasonography was suspected to be the enlarged cervix noted during the abdominal exploratory. The cervix is often seen as an oblique, hyperechoic, linear structure with ultrasonography in the longitudinal view and is usually slightly larger than the uterine diameter measuring 1.5–2 cm in length and 0.8 cm in diameter [10, 11], with the two structures blending into one another making it difficult to differentiate from the uterine body [9–11]. The cervix is often best visualized under hormonal (estrogen or progesterone) influence rather than during anestrus [11].

A surgical exploratory was elected and general anesthesia was induced with administration of fentanyl (5.0 mcg/kg, IV) and propofol (4 mg/kg, IV) to effect. An endotracheal tube was placed, and anesthesia was maintained with delivery of isoflurane in oxygen, with a constant rate infusion (CRI) of fentanyl (3–5 mcg/kg/hr, IV) for analgesia. After standard aseptic preparation for surgery, and with the patient positioned in dorsal recumbency, a caudal ventral midline incision was made for exploration of the caudal abdomen.

**Figure 3:**
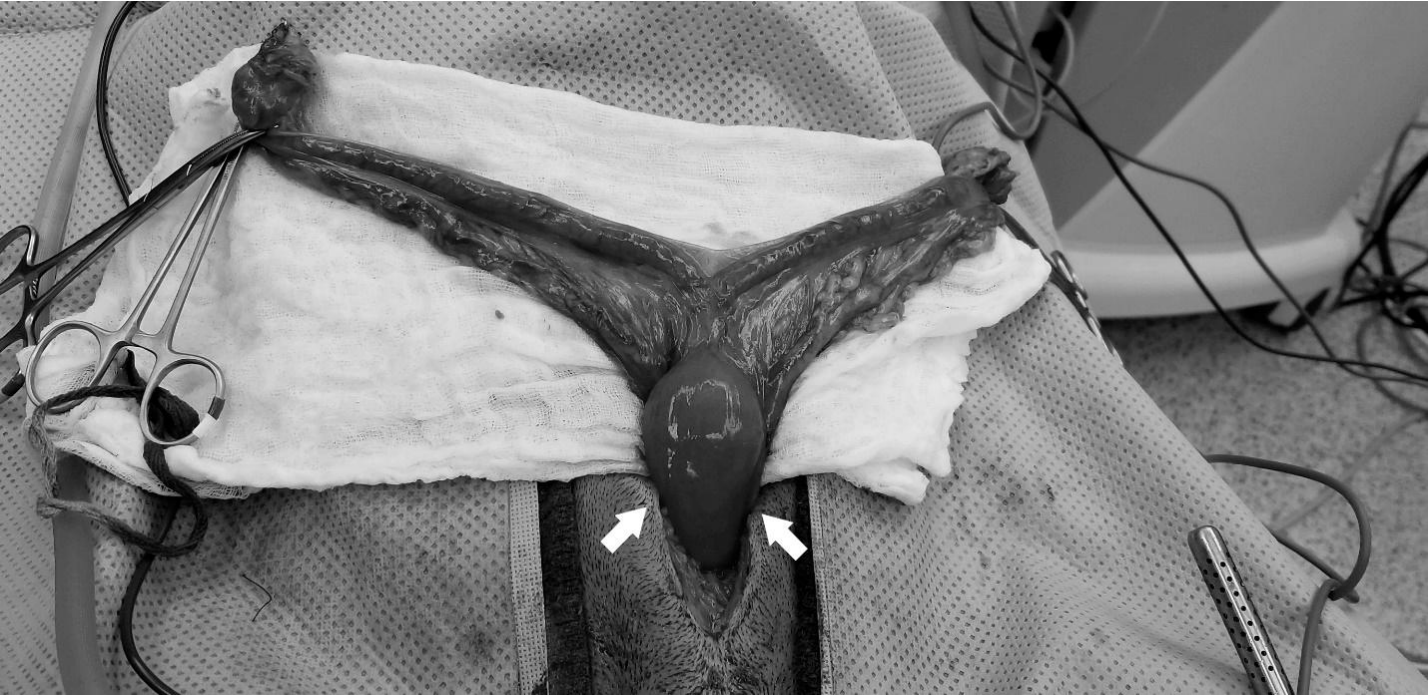
Intraoperative image of the ovaries, uterine horns and body, and cranial aspect of the vagina exteriorized from the abdominal cavity. Note the markedly thickened, edematous vaginal tissue (arrows).

Exploration of the caudal abdomen revealed the uterine horns to be normal and visible just beneath the body wall. The left and right ovarian pedicles and sections of the broad ligament were ligated and cauterized, allowing for the uterine horns and uterine body to be exteriorized. Further assessment of the reproductive tract revealed a normal uterine body and palpable swelling present on the dorsal aspect of the cervix, and distal to that, the vaginal tissue was markedly thickened and edematous (Figure 3). The cervix and vagina were retracted out of the abdominal cavity as far cranially as possible. Two Doyen intestinal clamps were placed over the vaginal body 2 cm caudal to the cervix over the vaginal body, approximately 1 cm apart from one another. The vaginal arteries were each ligated with circumferential ligatures using monofilament absorbable suture (size 3–0 polydioxanone^(a. PDS II, Ethicon, Johnson and Johnson, Somerville, NJ 08876)^) at the level of the vaginal body between the previously placed clamps. The vaginal body was transected between the clamps, distal to the ligatures, and a cut and oversew technique with monofilament absorbable suture (size 2–0 polydioxanone^(a. PDS II, Ethicon, Johnson and Johnson, Somerville, NJ 08876)^) in a simple continuous pattern was performed across the transected end of the vaginal body. The vaginal stump was checked for leakage, none of which was noted, then lavaged copiously with sterile saline and released back into the caudal abdominal cavity. Further exploration of the caudal abdomen was found to be unremarkable. The abdomen was lavaged copiously with sterile saline and the abdominal incision was closed in a routine fashion. Postoperatively, the dog was treated with amoxicillin-clavulanic acid (20 mg/kg, PO, *q* 12 hr) for 14 days for suspected vaginal infection. Pain was managed postoperatively with a fentanyl CRI (2–5 mcg/kg/hr, IV) that was tapered over 12 hours of hospitalization.

The ovaries, uterus, cervix, and vaginal tissue were submitted for histopathology. Examination of the ovaries revealed numerous follicles in various stages of differentiation present within the ovarian cortex such as several large hemorrhagic ovarian follicles (corpora hemorrhagica). Examination of the uterus revealed focally extensive weakening of the uterine epithelium with low numbers of lymphocytes, plasma cells, and neutrophils infiltrating the superficial lamina propria within the endometrium immediately adjacent to the cervix. The endometrium of the uterine horns (more cranial to the cervix) was found to be unremarkable. Examination of the vaginal tissue adjacent to the cervix found the vaginal epithelium to be moderately thickened and composed of stratified squamous keratinizing cells. The vaginal submucosal lymphatics were dilated and there was regionally extensive marked edema of the outer muscular layer and serosa with diffusely scattered neutrophils and macrophages throughout the edematous stroma, which extended to the surgical margins. There was focally extensive erosion and ulceration with low to moderate numbers of neutrophils on the surface. Based on the results of histopathology, the dog was diagnosed with mild to moderate regionally extensive subacute neutrophilic cervicovaginitis with mural edema and minimal locally extensive endometritis. The swelling is attributed to edema particularly within the deep, outer layers of the vagina, associated with suppurative vaginitis. The neutrophilic infiltrate and character of the discharge suggested bacterial vaginitis, but no infectious organisms were seen. Nondegenerate neutrophils are often visualized in large quantities when a dog is in diestrus, whereas in the case of vaginal infection, many degenerate neutrophils are often noted which can occur in the presence or absence of bacteria [6]. There was minimal inflammation of the endometrium next to the cervix, interpreted as extension of cervicovaginitis. Uterine, cervical, and vaginal tissue were submitted for aerobic and anaerobic culture. No growth was noted with any of the cultures after one week.

The dog was transitioned from intravenous to oral analgesia (carprofen at 2.2 mg/kg, PO, *q* 12 hr). The vaginal discharge, fever, lethargy, and vomiting had resolved, and the dog was eating and subsequently was discharged 24 hours postoperatively. Follow-up information was obtained from a phone conversation with the dog's owners. The owners did not report any return of clinical signs related to vaginitis in the 6 months since surgery.

## 3. Discussion

Vaginitis, or inflammation of the vagina, is a rare disease in dogs that is generally considered to be primary or secondary [1–4, 6, 7]. Primary or uncomplicated vaginitis is relatively uncommon, but is usually bacterial in origin such as *Brucella canis *or* Mycoplasma* spp, with fungal and viral (canine herpes virus) vaginitis being less common [1, 2, 5, 12, 13]. Secondary vaginitis is relatively common and usually occurs in spayed or intact bitches and is often due to anatomical or structural abnormalities. The most common type of structural abnormality is a vaginal stricture which usually occurs cranial to the vaginal papilla near the vestibulovaginal junction [2, 5]. Vaginal strictures or septa can be detected by digital vaginal examination in 88% of cases [14, 15]. Poor perineal conformation such as a “hooded vulva” can also lead to secondary vaginitis, and although this patient's vulva was enlarged, the conformation was appropriate [2]. A digital vaginal examination was performed in the described case and no structural abnormalities were palpable. Urinary tract infections and vaginitis have also been seen concurrently as infected urine passing through the vagina could predispose a dog to vaginitis [16]. This dog had no clinical signs related to the lower urinary tract beyond the vaginal discharge, and the urinary tract was grossly normal, so urine was not collected for urinalysis and culture. While a urinary tract infection cannot be ruled out as primary cause, the patient's focal disease makes the differential seem less likely, as does the complete resolution of signs postoperatively. Other causes for secondary vaginitis include trauma, foreign bodies, and vaginal masses [1–4, 7], none of which were found in the case presented.

Vaginitis can also be subdivided into forms regarding the patient's age, which include juvenile or puppy vaginitis and adult-onset vaginitis, but it is important to note that this disease can occur in any age, breed, or ovarian condition [2, 4]. Juvenile vaginitis is typically associated with bitches that have not yet undergone puberty with most affected patients exhibiting no clinical signs [2], and this dog had experienced its first heat cycle. Inflammation of the vestibule and vagina in the adult bitch usually is caused by some predisposing condition resultant of anatomic anomalies that are not identified or are difficult to correct, and although idiopathic adult-onset vaginitis can occur, it is often present in spayed females [4, 5, 13]. Based on the literature, this is a rare presentation of vaginitis.

Clinical signs associated with vaginitis generally include vulvar swelling, vulva licking, pollakiuria, urinary incontinence and mucoid, mucopurulent, and less commonly blood or blood-tinged vaginal discharge [1–6]; however, this dog presented with a fever, lethargy, abdominal pain, and brown vaginal discharge. Pyometra has been documented as a differential diagnosis for dogs experiencing vaginitis, which was initially suspected in this case due to the clinical signs and tubular fluid-filled structure in the caudal abdomen noted on ultrasound [1]. The dog described presented with moderate neutropenia and was febrile, which is not a typical presentation for vaginitis, but the source did not appear to be uterine as the uterine horns and majority of the uterine body were noted to be grossly and histopathologically normal on examination. There is a paucity in the literature describing cervicovaginitis of this nature, and it could be postulated that the cervix became involved due to ascension of bacterial vaginitis. Aerobic and anaerobic bacterial cultures were negative, though the dog had been administered antibiotics for approximately 36 hours prior to presentation. Cytologic examination of the vaginal epithelium in dogs with vaginitis reveals nonkeratinized epithelial cells, which was not consistent with the keratinized stratified epithelium found in this dog, though it is likely this difference is due to her intact status and recent estrus [2, 3].

Treatment of vaginitis in an affected dog depends on the form present. In the case of juvenile vaginitis, most cases resolve spontaneously with time [2]. In adult-onset vaginitis, most cases resolve once the predisposing problem has been corrected. The most common primary problems reported are urinary tract infections in 2–60% of cases, vaginal anatomical anomalies in 20–36% of cases, and systemic disease in 15% of cases [1–3, 5]. In most cases, vaginitis resolves regardless of therapy as it is generally self-limiting [4, 8]. However, this case of severe cervicovaginitis resulted in significant systemic effects and required surgical intervention for resolution.

While medical therapy or benign neglect is the typical approach regarding the treatment of vaginitis, surgical intervention has been reported. Subtotal vaginectomy has been described as treatment for a dog with severe generalized chronic ulcerative vaginitis [8], but this procedure is often most indicated in cases of vaginal neoplasia and extensive vaginal disease [8, 17, 18] that is more than 2 cm cranial to the vestibulovaginal junction [19]. Subtotal vaginectomy is often performed using a combined ventral midline celiotomy and episiotomy with or without pelvic osteotomy/ostectomy to allow for compartmental resection of the vagina from the cervix to the vestibulovaginal junction [8, 19]. Interestingly, in the case reported here, the diseased tissues extended well-cranial to the vagina and included the entire cervix and caudal uterine body. Typical treatment of vaginitis would not have been sufficient for resolution of the disease in this dog experiencing systemic illness secondary to the condition.

In the present case, the patient had a history of beginning her first heat cycle 10 days prior to presentation which started as hemorrhagic, odorless vaginal discharge that progressed to brown, malodorous discharge. Histopathologic examination of the ovaries revealed various follicular cycles including corpus hemorrhagica denoting recent ovulation and supporting the history of recent estrus. It is a valid consideration to believe that the patient's recent estrus cycle incited regional inflammation within the vagina causing subsequent cervicovaginitis, although to the authors' knowledge this has not been reported. The pathophysiology of vaginitis is still poorly understood, so there may be an unknown underlying cause for vaginitis in the patient described. This report illustrates the benefit of using abdominal ultrasonography in supporting the diagnosis of cervicovaginitis based on suggestive clinical signs and signalment. In addition, we document ovariohysterectomy and partial vaginectomy as viable treatment options with minimal postoperative and long-term complications for the management of unusually severe subacute onset of suppurative cervicovaginitis with mild metritis in a dog.
